# Editorial: Pharmacogenomics of Adverse Drug Reactions

**DOI:** 10.3389/fgene.2022.859909

**Published:** 2022-03-07

**Authors:** Moneeza K. Siddiqui, Jasmine Luzum, Marieke Coenen, Seyed Hamidreza Mahmoudpour

**Affiliations:** ^1^ Division of Population Health & Genomics, Pat McPherson Centre for Pharmacogenetics & Pharmacogenomics, School of Medicine, Ninewells Hospital & Medical School, University of Dundee, Dundee, United Kingdom; ^2^ Department of Clinical Pharmacy, University of Michigan College of Pharmacy, Ann Arbor, MI, United States; ^3^ Department of Human Genetics, Radboud University Medical Center, Radboud Institute for Health Sciences, Nijmegen, Netherlands; ^4^ Department of Biometry and Bioinformatics, Institute for Medical Biostatistics, Epidemiology, and Informatics (IMBEI), University Medical Center Mainz, Mainz, Germany

**Keywords:** pharmacogenomics, adverse drug reactions (ADRs), pharmacogenetics, personalized medicine, adverse (side) effects

## Why Do Adverse Drug Reactions Matter

Therapeutic adherence is necessary to achieve drug targets, improve outcomes, and reduce the occurrence of morbidity and preventable mortality. Adverse drug reactions (ADRs) are a major contributor to reduced compliance, adherence to treatments, morbidity, and mortality. ADRs are estimated to be the fourth leading cause of death in the United States—ahead of pulmonary disease (before the COVID-19 pandemic), diabetes, AIDS, pneumonia, accidents, and automobile deaths (Center for Drug Evaluation and Research, 2021). Individuals experiencing ADRs are more likely to seek follow up visits with healthcare providers, have decreased compliance, and a higher rate of drug failure. Therefore, understanding both the clinical and genetic risk factors for ADR is crucial in the delivery of precision medicine.

## Data substrates for ADR Research

Randomised clinical trials provide a key resource for post-hoc pharmacogenetic studies. However, there is a substantial diversion between in-trial behaviours and real-world practices. RCTs usually have strict inclusion and exclusion criteria, meaning that the patients enrolled in RCTs typically do not represent the complexity of patients in real-world clinical practice. Moreover, the practice of conducting a run-in period results in the retention of a sub-set of individuals more likely to comply with therapy. RCTs are also typically run for a finite amount of time, typically one to a few years at the most, during which dose and therapies are not changed, and patients in the real-world are treated with drugs for much longer periods of time. On the other hand, observational data resources reflect real-world behaviours such as highly complex patients with multiple drug therapies and comorbidities, physicians adapting therapies based on drug response, and altered prescribing patterns due to changes in guidelines or expiring patents. In observational data resources, the longer follow up period allows researchers to better gauge patterns of adherence, and adverse drug reactions that are milder but perhaps more commonplace. While RCTs are the most reliable source of recorded severe ADRs, individual behaviours in the ambulatory setting shed light on long-term adherence, drug-response, and milder ADRS. This is reflected in the breadth of articles in this special issue, which are all based on observational data resources from across the world, including the United Kingdom, United States, India, and China.

## What Does Pharmacogenetics Add?

Pharmacogenetics and genomics allow researchers to assess the role of genetic variants in key genes such as those involved in pharmacokinetic and immune pathways. This can shed light on the functional role of variants and help tailor newer therapies. There are several examples in which candidate gene studies have identified and validated genetic variants associated with ADRs, which are now recommended in clinical practice guidelines and by regulatory agencies across the world to reduce patient risk of ADRs (PharmGKB, 2022). However, ADRs remain a major clinical problem, and thus more real-world evidence is needed. While a lot of studies have focussed on candidate gene studies, latterly research has suggested that most genome-wide signals are found in non-candidate regions–further highlighting the potential for discovery in the field of pharmacogenetics (Linskey et al., 2021). Another avenue of investigation are rare variants, which are poorly characterised due to the lack of sequencing-based genomic data. Recent studies have highlighted the added value of rare variants in drug response, and it stands to reason that they would have value in understanding ADRs (McInnes et al., 2021). The majority of pharmacogenetic research has also focused on patients of European ancestry; this is largely driven by the preponderance of trials and observational cohorts available on individuals of European descent. However, this leaves a substantial gap in our understanding of ADRs and pharmacogenetics in non-European ethnicities, a field with enormous potential.

## In This Special Issue

The special issue advances the much-needed evidence base for pharmacogenetics of adverse drug reactions by covering a wide breadth of therapeutic areas, including cardiovascular, neurology, endocrinology and diabetes, as well as cancer. For clinical conditions with limited treatment strategies and with severe adverse outcomes, pharmacogenetics helps identify new drug targets and patients at risk of ADRs. In this issue, Tirozzi and Willcocks investigate ADRs in Parkinson’s disease and schizophrenia that present such challenges (Tirozzi et al.; Willcocks et al.). This special issue also advances the evidence base for non-European populations such as East Asian and Asian Indians bringing a much needed global perspective (Shanbhag et al.). Evidence is also emerging to show that genetic risk for ADRs is polygenic, and the papers by Melhem et al. and Ooi et al. in this special issue applied advanced methods such as genetic scores and machine learning to further our understanding of the polygenic risk for ADRs (Melhem et al.; Ooi et al.). Moving forward, the field of pharmacogenetics and genomics for adverse drug reactions has three main lacunae: the use of data from under-represented ethnicities, methods to translate genetic risk to clinical practice, and the use of novel genetic methods that can help establish causal relationships such as Mendelian Randomisation.
